# Zirconium(IV) dilanthanum(III) penta­sulfide

**DOI:** 10.1107/S1600536811045193

**Published:** 2011-11-05

**Authors:** Adam D. Raw, James A. Ibers

**Affiliations:** a Department of Chemistry, Northwestern University, 2145 Sheridan Rd, Evanston, IL 60208-3113, USA

## Abstract

Zirconium(IV) dilanthanum(III) penta­sulfide, ZrLa_2_S_5_, crystallizes with four formula units in the space group *Pnma* in the U_3_S_5_ structure type. The asymmetric unit comprises one Zr, one La and four S atoms. The Zr and three S atoms are situated on mirror planes. The structure consists of LaS_8_ face-sharing bicapped distorted trigonal prisms and ZrS_7_ edge-sharing monocapped octa­hedra.

## Related literature

The cell parameters of ZrLa_2_S_5_ were previously reported from X-ray powder diffraction measurements (Kokhno & Sere­brennikov, 1977 ▶). In a separate study, single-crystal X-ray diffraction measurements were used to determine the lattice parameters but not the structural parameters (Donohue & Jeitschko, 1974 ▶). Given that these lattice parameters, the space group, and the stoichiometry are similar to those of U_3_S_5_, it was assumed that ZrLa_2_S_5_ and U_3_S_5_ are isotypic (Donohue & Jeitschko, 1974 ▶). For analogous structures, see: Du Pont de Nemours (1976 ▶) and Potel *et al.* (1972 ▶). Physical property measurements of this and related compounds have been reported. For optical properties, see: Alekseeva *et al.* (1980 ▶); for electrical properties, see: Senova *et al.* (1984 ▶). For synthetic details, see: Jin *et al.* (2009 ▶). For ionic radii, see: Shannon (1976 ▶). For standardization of structural data, see: Gelato & Parthé (1987 ▶).

## Experimental

### 

#### Crystal data

ZrLa_2_S_5_
*M* *_r_* = 529.34
Orthorhombic, 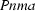

*a* = 11.4784 (4) Å
*b* = 8.2010 (3) Å
*c* = 7.3799 (3) Å
*V* = 694.70 (5) Å^3^
*Z* = 4
Mo *K*α radiation
μ = 14.93 mm^−1^
*T* = 100 K
0.12 × 0.11 × 0.09 mm

#### Data collection

Bruker APEXII CCD diffractometer
Absorption correction: numerical [face indexed (*SADABS*; Bruker, 2009 ▶)] *T*_min_ = 0.263, *T*_max_ = 0.340
8134 measured reflections
890 independent reflections
877 reflections with *I* > 2σ(*I*)
*R*_int_ = 0.018

#### Refinement

*R*[*F* ^2^ > 2σ(*F* ^2^)] = 0.017
*wR*(*F* ^2^) = 0.056
*S* = 2.51
890 reflections
44 parameters
Δρ_max_ = 2.20 e Å^−3^
Δρ_min_ = −0.54 e Å^−3^

### 

Data collection: *APEX2* (Bruker, 2009 ▶); cell refinement: *SAINT* (Bruker, 2009 ▶); data reduction: *SAINT*; program(s) used to solve structure: *SHELXS97* (Sheldrick, 2008 ▶); program(s) used to refine structure: *SHELXL97* (Sheldrick, 2008 ▶); molecular graphics: *CrystalMaker* (Palmer, 2009 ▶); software used to prepare material for publication: *SHELXL97*.

## Supplementary Material

Crystal structure: contains datablock(s) I, global. DOI: 10.1107/S1600536811045193/wm2549sup1.cif

Structure factors: contains datablock(s) I. DOI: 10.1107/S1600536811045193/wm2549Isup2.hkl

Additional supplementary materials:  crystallographic information; 3D view; checkCIF report

## Figures and Tables

**Fig. 1.**
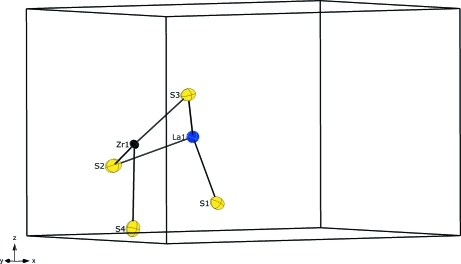
The asymmetric unit of ZrLa2S5. Displacement ellipsoids are displayed at the 95% probability level.

**Fig. 2.**
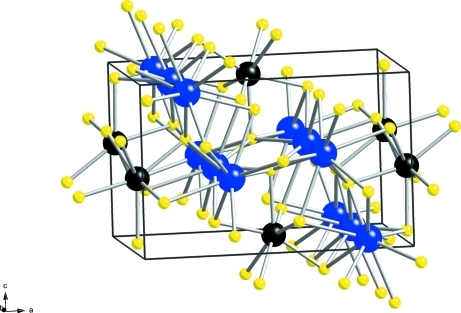
The ZrLa2S5 structure. La atoms are blue, Zr atoms are black, S atoms are yellow

## supplementary crystallographic information

### Comment

Single crystals of ZrLa_2_S_5_ resulted from attempts to synthesize zirconium analogues of the uranium lanthanide oxysulfide compound UYb_2_O_2_S_3_ (Jin *et al.*, 2009).

ZrLa_2_S_5_ adopts the U_3_S_5_ structure type (Potel *et al.*, 1972). The unit-cell dimensions have been previously reported from single-crystal X-ray diffraction data (Donohue & Jeitschko, 1974) and from powder X-ray diffraction data (Kokhno & Serebrennikov, 1977). Unit-cell dimensions for ZrLa_2_S_5_ from the two single-crystal determinations compare favorably: *a* = 11.4864 (5), *b* = 8.2167 (5), *c* = 7.3894 (3) Å at room temperature (Donohue & Jeitschko, 1974) *versus a* = 11.4784 (4), *b* = 8.2010 (3), *c* = 7.3799 (3) Å from the present study at 100 K. (See also Kokhno & Serebrennikov, 1977). Isostructural compounds have been previously reported based on X-ray powder diffraction measurements for all trivalent lanthanides and yttrium excluding promethium, europium and ytterbium (Du Pont de Nemours, 1976).

The La—S interatomic distances (Table 1, Fig. 1) in the face-sharing bicapped distorted trigonal prisms LaS_8_ (Fig. 2) range from 2.8861 (8) to 3.0698 (9) Å. The Zr—S distances range from 2.5704 (8) to 2.7421 (11) Å. These values are close to the distances of 3.00 Å for La—S and 2.62 Å for Zr—S calculated from the summed ionic radii (Shannon, 1976).

Physical property measurements of ZrLa_2_S_5_ have been reported by Alekseeva *et al.* (1980) and Senova *et al.* (1984).

### Experimental

ZrO_2_ (99.99%, Aldrich) and La_2_S_3_ (99.9%, Strem) were used as received. Sb_2_S_3_ was synthesized from the elements. ZrLa_2_S_5_ was crystallized in a two step reaction in carbon-coated fused-silica tubes that had been evacuated to 10 ^-4^ Torr. In the first step, 0.02 g (0.16 mmol) ZrO_2_ and 0.3 g (0.08 mmol) La_2_S_3_ were heated at 1273 K for 99 h and cooled to 298 K in 14 h. The resulting powder was combined with 0.02 g (0.06 mmol) Sb_2_S_3_ and heated at 1273 K for 99 h then cooled to 873 K at a rate of 2 K/h before cooling to 298 K over 10 h. The resulting ZrLa_2_S_5_ formed black prismatic crystals in low yield (<5 wt%). These were mechanically separated from the remaining powder.

### Refinement

The atomic positions were standardized with use of the program *STRUCTURE TIDY* (Gelato & Parthé, 1987). The highest peak of 2.2 (2) e/Å^3^ is 0.50 Å and the deepest hole of -0.5 (2) e/Å^3^ is 0.99 Å from the Zr position.

### Crystal data

**Table:**

ZrLa_2_S_5_	*D*_x_ = 5.061 Mg m^−^^3^
*M**_r_* = 529.34	Mo *K*α radiation, λ = 0.71073 Å
Orthorhombic, *P**n**m**a*	Cell parameters from 6255 reflections
*a* = 11.4784 (4) Å	θ = 3.1–27.9°
*b* = 8.2010 (3) Å	µ = 14.93 mm^−^^1^
*c* = 7.3799 (3) Å	*T* = 100 K
*V* = 694.70 (5) Å^3^	Prism, black
*Z* = 4	0.12 × 0.11 × 0.09 mm
*F*(000) = 936	

### Data collection

**Table:**

Bruker APEXII CCD diffractometer	890 independent reflections
Radiation source: fine-focus sealed tube	877 reflections with *I* > 2σ(*I*)
graphite	*R*_int_ = 0.018
φ and ω scans	θ_max_ = 27.9°, θ_min_ = 3.3°
Absorption correction: numerical [face indexed (*SADABS*; Bruker, 2009)]	*h* = −14→15
*T*_min_ = 0.263, *T*_max_ = 0.340	*k* = −10→6
8134 measured reflections	*l* = −9→9

### Refinement

**Table:**

Refinement on *F*^2^	Primary atom site location: structure-invariant direct methods
Least-squares matrix: full	Secondary atom site location: difference Fourier map
*R*[*F*^2^ > 2σ(*F*^2^)] = 0.017	[1.00000 + 0.00000exp(0.00(sinθ/λ)^2^)]/ [σ^2^(*F*_o_^2^) + 0.0000 + 0.0000**P* + (0.0158*P*)^2^ + 0.0000sinθ/λ] where *P* = 1.00000*F*_o_^2^ + 0.00000*F*_c_^2^
*wR*(*F*^2^) = 0.056	(Δ/σ)_max_ = 0.001
*S* = 2.51	Δρ_max_ = 2.20 e Å^−^^3^
890 reflections	Δρ_min_ = −0.54 e Å^−^^3^
44 parameters	Extinction correction: *SHELXL97* (Sheldrick, 2008), Fc^*^=kFc[1+0.001xFc^2^λ^3^/sin(2θ)]^-1/4^
0 restraints	Extinction coefficient: 0.0015 (2)

### Special details

**Table:**

Geometry. All e.s.d.'s (except the e.s.d. in the dihedral angle between two l.s. planes) are estimated using the full covariance matrix. The cell e.s.d.'s are taken into account individually in the estimation of e.s.d.'s in distances, angles and torsion angles; correlations between e.s.d.'s in cell parameters are only used when they are defined by crystal symmetry. An approximate (isotropic) treatment of cell e.s.d.'s is used for estimating e.s.d.'s involving l.s. planes.
Refinement. Refinement of *F*^2^ against ALL reflections. The weighted *R*-factor *wR* and goodness of fit *S* are based on *F*^2^, conventional *R*-factors *R* are based on *F*, with *F* set to zero for negative *F*^2^. The threshold expression of *F*^2^ > σ(*F*^2^) is used only for calculating *R*-factors(gt) *etc*. and is not relevant to the choice of reflections for refinement. *R*-factors based on *F*^2^ are statistically about twice as large as those based on *F*, and *R*- factors based on ALL data will be even larger.

### Fractional atomic coordinates and isotropic or equivalent isotropic displacement parameters (Å^2^)

**Table:**

	*x*	*y*	*z*	*U*_iso_*/*U*_eq_	
La1	0.328759 (18)	0.501789 (17)	0.44160 (3)	0.00537 (12)	
Zr1	0.01001 (3)	0.2500	0.42914 (5)	0.00286 (13)	
S1	0.29147 (10)	0.2500	0.15995 (15)	0.0061 (2)	
S2	0.07363 (7)	0.53474 (10)	0.32234 (11)	0.00812 (18)	
S3	0.19311 (10)	0.2500	0.64058 (15)	0.0063 (2)	
S4	0.00443 (9)	0.2500	0.05768 (14)	0.0073 (2)	

### Atomic displacement parameters (Å^2^)

**Table:**

	*U*^11^	*U*^22^	*U*^33^	*U*^12^	*U*^13^	*U*^23^
La1	0.00643 (17)	0.00439 (17)	0.00529 (17)	0.00023 (5)	−0.00022 (6)	−0.00036 (5)
Zr1	0.0030 (2)	0.0028 (2)	0.0028 (2)	0.000	−0.00063 (13)	0.000
S1	0.0073 (5)	0.0053 (5)	0.0056 (5)	0.000	−0.0013 (4)	0.000
S2	0.0102 (4)	0.0080 (3)	0.0062 (4)	0.0004 (3)	0.0007 (3)	−0.0005 (3)
S3	0.0086 (5)	0.0051 (5)	0.0051 (5)	0.000	0.0008 (4)	0.000
S4	0.0066 (6)	0.0062 (5)	0.0090 (6)	0.000	0.0007 (4)	0.000

### Geometric parameters (Å, °)

**Table:**

La1—S4^i^	2.8861 (8)	Zr1—S2^viii^	2.7206 (9)
La1—S4^ii^	2.9230 (7)	Zr1—S2^ix^	2.7206 (9)
La1—S1^ii^	2.9402 (8)	Zr1—S4	2.7421 (11)
La1—S1	2.9610 (8)	S1—Zr1^i^	2.5932 (12)
La1—S3	2.9740 (8)	S1—La1^x^	2.9402 (8)
La1—S3^iii^	3.0235 (8)	S1—La1^iii^	2.9402 (8)
La1—S2^ii^	3.0398 (8)	S1—La1^vi^	2.9609 (8)
La1—S2	3.0698 (9)	S2—Zr1^viii^	2.7206 (9)
La1—La1^iv^	4.0247 (4)	S2—La1^iii^	3.0398 (8)
La1—La1^v^	4.0712 (3)	S3—La1^vi^	2.9739 (8)
La1—La1^iii^	4.1092 (2)	S3—La1^ii^	3.0235 (8)
La1—La1^ii^	4.1092 (2)	S3—La1^xi^	3.0235 (8)
Zr1—S2^vi^	2.5704 (8)	S4—La1^xii^	2.8861 (8)
Zr1—S2	2.5705 (8)	S4—La1^vii^	2.8861 (8)
Zr1—S1^vii^	2.5932 (12)	S4—La1^iii^	2.9230 (7)
Zr1—S3	2.6176 (12)	S4—La1^x^	2.9230 (7)
			
S4^i^—La1—S4^ii^	92.295 (9)	S2—La1—La1^ii^	80.696 (17)
S4^i^—La1—S1^ii^	145.31 (3)	La1^iv^—La1—La1^ii^	103.733 (7)
S4^ii^—La1—S1^ii^	70.41 (3)	La1^v^—La1—La1^ii^	90.408 (4)
S4^i^—La1—S1	66.63 (3)	La1^iii^—La1—La1^ii^	127.785 (11)
S4^ii^—La1—S1	141.82 (3)	S2^vi^—Zr1—S2	130.58 (4)
S1^ii^—La1—S1	143.100 (13)	S2^vi^—Zr1—S1^vii^	101.36 (2)
S4^i^—La1—S3	82.42 (2)	S2—Zr1—S1^vii^	101.36 (2)
S4^ii^—La1—S3	132.98 (3)	S2^vi^—Zr1—S3	87.40 (2)
S1^ii^—La1—S3	87.96 (2)	S2—Zr1—S3	87.40 (2)
S1—La1—S3	77.69 (2)	S1^vii^—Zr1—S3	158.10 (3)
S4^i^—La1—S3^iii^	122.76 (3)	S2^vi^—Zr1—S2^viii^	153.69 (2)
S4^ii^—La1—S3^iii^	78.51 (2)	S2—Zr1—S2^viii^	73.59 (3)
S1^ii^—La1—S3^iii^	84.12 (2)	S1^vii^—Zr1—S2^viii^	80.19 (3)
S1—La1—S3^iii^	86.66 (2)	S3—Zr1—S2^viii^	83.19 (3)
S3—La1—S3^iii^	141.885 (17)	S2^vi^—Zr1—S2^ix^	73.59 (3)
S4^i^—La1—S2^ii^	70.75 (3)	S2—Zr1—S2^ix^	153.69 (2)
S4^ii^—La1—S2^ii^	63.66 (2)	S1^vii^—Zr1—S2^ix^	80.19 (3)
S1^ii^—La1—S2^ii^	74.61 (3)	S3—Zr1—S2^ix^	83.19 (3)
S1—La1—S2^ii^	129.30 (2)	S2^viii^—Zr1—S2^ix^	80.92 (4)
S3—La1—S2^ii^	70.62 (3)	S2^vi^—Zr1—S4	72.55 (2)
S3^iii^—La1—S2^ii^	140.91 (2)	S2—Zr1—S4	72.55 (2)
S4^i^—La1—S2	136.89 (2)	S1^vii^—Zr1—S4	73.97 (3)
S4^ii^—La1—S2	130.36 (2)	S3—Zr1—S4	127.93 (4)
S1^ii^—La1—S2	69.42 (3)	S2^viii^—Zr1—S4	131.74 (2)
S1—La1—S2	73.87 (3)	S2^ix^—Zr1—S4	131.74 (2)
S3—La1—S2	72.74 (3)	Zr1^i^—S1—La1^x^	108.37 (3)
S3^iii^—La1—S2	69.57 (3)	Zr1^i^—S1—La1^iii^	108.37 (3)
S2^ii^—La1—S2	128.71 (2)	La1^x^—S1—La1^iii^	87.63 (3)
S4^i^—La1—La1^iv^	46.526 (16)	Zr1^i^—S1—La1^vi^	92.19 (3)
S4^ii^—La1—La1^iv^	45.769 (15)	La1^x^—S1—La1^vi^	88.267 (9)
S1^ii^—La1—La1^iv^	110.25 (2)	La1^iii^—S1—La1^vi^	159.29 (4)
S1—La1—La1^iv^	106.65 (2)	Zr1^i^—S1—La1	92.19 (3)
S3—La1—La1^iv^	113.61 (2)	La1^x^—S1—La1	159.29 (4)
S3^iii^—La1—La1^iv^	104.09 (2)	La1^iii^—S1—La1	88.267 (9)
S2^ii^—La1—La1^iv^	56.031 (16)	La1^vi^—S1—La1	88.43 (3)
S2—La1—La1^iv^	173.643 (17)	Zr1—S2—Zr1^viii^	106.41 (3)
S4^i^—La1—La1^v^	135.682 (15)	Zr1—S2—La1^iii^	107.38 (3)
S4^ii^—La1—La1^v^	45.861 (14)	Zr1^viii^—S2—La1^iii^	144.80 (3)
S1^ii^—La1—La1^v^	46.186 (15)	Zr1—S2—La1	95.92 (3)
S1—La1—La1^v^	134.217 (15)	Zr1^viii^—S2—La1	101.56 (3)
S3—La1—La1^v^	133.973 (16)	La1^iii^—S2—La1	84.53 (2)
S3^iii^—La1—La1^v^	47.682 (15)	Zr1—S3—La1^vi^	97.24 (3)
S2^ii^—La1—La1^v^	95.655 (16)	Zr1—S3—La1	97.24 (3)
S2—La1—La1^v^	84.950 (15)	La1^vi^—S3—La1	87.95 (3)
La1^iv^—La1—La1^v^	90.417 (4)	Zr1—S3—La1^ii^	111.80 (3)
S4^i^—La1—La1^iii^	107.70 (2)	La1^vi^—S3—La1^ii^	150.89 (4)
S4^ii^—La1—La1^iii^	123.806 (19)	La1—S3—La1^ii^	86.491 (11)
S1^ii^—La1—La1^iii^	106.89 (2)	Zr1—S3—La1^xi^	111.80 (3)
S1—La1—La1^iii^	45.659 (16)	La1^vi^—S3—La1^xi^	86.491 (11)
S3—La1—La1^iii^	102.01 (2)	La1—S3—La1^xi^	150.89 (4)
S3^iii^—La1—La1^iii^	46.251 (17)	La1^ii^—S3—La1^xi^	84.64 (3)
S2^ii^—La1—La1^iii^	172.533 (17)	Zr1—S4—La1^xii^	90.83 (3)
S2—La1—La1^iii^	47.425 (16)	Zr1—S4—La1^vii^	90.83 (3)
La1^iv^—La1—La1^iii^	128.467 (9)	La1^xii^—S4—La1^vii^	91.36 (3)
La1^v^—La1—La1^iii^	90.409 (4)	Zr1—S4—La1^iii^	106.12 (3)
S4^i^—La1—La1^ii^	107.50 (2)	La1^xii^—S4—La1^iii^	163.03 (4)
S4^ii^—La1—La1^ii^	91.72 (2)	La1^vii^—S4—La1^iii^	87.705 (9)
S1^ii^—La1—La1^ii^	46.074 (16)	Zr1—S4—La1^x^	106.12 (3)
S1—La1—La1^ii^	124.18 (2)	La1^xii^—S4—La1^x^	87.705 (9)
S3—La1—La1^ii^	47.258 (17)	La1^vii^—S4—La1^x^	163.03 (4)
S3^iii^—La1—La1^ii^	128.91 (2)	La1^iii^—S4—La1^x^	88.28 (3)
S2^ii^—La1—La1^ii^	48.044 (16)		

Symmetry codes: (i) *x*+1/2, *y*, −*z*+1/2; (ii) −*x*+1/2, −*y*+1, *z*+1/2; (iii) −*x*+1/2, −*y*+1, *z*−1/2; (iv) −*x*+1, −*y*+1, −*z*+1; (v) *x*, −*y*+3/2, *z*; (vi) *x*, −*y*+1/2, *z*; (vii) *x*−1/2, *y*, −*z*+1/2; (viii) −*x*, −*y*+1, −*z*+1; (ix) −*x*, *y*−1/2, −*z*+1; (x) −*x*+1/2, *y*−1/2, *z*−1/2; (xi) −*x*+1/2, *y*−1/2, *z*+1/2; (xii) *x*−1/2, −*y*+1/2, −*z*+1/2.
